# Physiological aging in India: The role of the epidemiological transition

**DOI:** 10.1371/journal.pone.0287259

**Published:** 2023-07-19

**Authors:** Astrid Krenz, Holger Strulik

**Affiliations:** 1 Department of Management and Economics, Center for Entrepreneurship, Innovation and Transformation (CEIT), Ruhr University Bochum, Bochum, Germany; 2 Department of Economics, University of Göttingen, Göttingen, Germany; VART Consulting PVT LTD, INDIA

## Abstract

We construct a cohort-based frailty index from age-related health deficits to investigate physiological aging in India over the period 1990-2019. During this period, the Indian states underwent at different speeds the epidemiological transition and experienced unprecedented economic growth. We show that the rate of physiological aging remained remarkably stable to the changing environment. Age-related health deficits increased by about 3 percent per year of age with little variation across states, ages, cohorts, and over time. We find that, with advancing epidemiological transition, health deficits for given age declined at the individual level (within states and within cohorts). Across cohorts born between 1900 and 1995, we show that, for given age, health deficits are higher for later-born cohorts until birth years around 1940 and remained trendless afterwards. We propose a selection-based theory of aging during the epidemiological transition that explains these facts.

## Introduction

From a demographer’s perspective, India is a relatively young country that is aging rapidly. The old age dependency ratio (population share age 65+ over population share 20 to 64) increased from 7.9 percent in 1990 to 11.7 percent in 2022 and is predicted to increase further to 22.5 percent in 2050 [1]. Several scholars have discussed the potential economic, social, and health challenges caused by India’s population aging [2, 3]. Most of these challenges, however, do not directly follow from the chronological aging of individuals but from their physiological aging, understood as the “intrinsic, cumulative, progressive, and deleterious loss of function that eventually culminates in death” [4]. The distinction is important and policy-relevant because physiological aging is malleable while chronological aging is immutable.

In this study, we investigate the physiological aging of the Indian population stratified by states, gender, age-groups, and cohorts over time. We measure physiological age by the frailty index, which is the proportion of potential aging-related (chronic) health deficits that are present in a person. The index has been developed by Mitnitski et al. [5] and has since been used in countless studies in gerontology, medical science, and, more recently, in economics (for reviews see [6–8]). We used Dalgaard et al.’s [9] adaptation of the frailty index to measure physiological aging at the group-level (of e.g. age-groups and cohorts). Using data provided by the India State-level Disease Burden Initiative (GBD-I, [10]) we constructed a frailty index from prevalence rates of 33 aging-related non-communicable diseases and built a panel data set comprising five-year age groups from ages 20–24 to 90–94 for the Indian states over the period 1990–2019 and cohorts born between 1900 and 1995.

During the observation period, the Indian states experienced an epidemiological transition with a great variety of levels and speeds of the transition. This feature allows (to the best of our knowledge, for the first time) to investigate how physiological aging is affected by the epidemiological transition. The original formulation of the epidemiological transition conceptualized it as a sequence of separate stages and, in particular, distinguished a stage of declining prevalence of communicable diseases followed by a stage of declining prevalence of non-communicable diseases [11]. Modern reformulations, however, refute the strict separation of distinct stages for communicable and non-communicable diseases and acknowledge that a decline of infections is accompanied by a simultaneous decline in chronic diseases [12]. For the U.S., for example, it has been shown that chronic diseases and functional limitations among older men decreased by over 60 percent from the early 20th century to the 1970s, and that a significant portion of the decrease was due to declining infections [13, 14]. An important pathway from reduced exposure to infections to less chronic diseases and slower aging runs through a slowdown of inflammaging, i.e. chronic inflammation [15–18]. From this mechanism, we would expect that people in states where the epidemiological transition has progressed faster would have, on average, a lower frailty index for a given chronological age. On the other hand, a lower lifetime exposure to infectious diseases increases survival probability, especially of individuals with pre-conditions. The selection (or survivorship) effect declines such that at any age more frail people survive and later-born cohorts may actually have a higher frailty index for a given age. These issues are explored below.

Our analysis begins with a validation of the constructed frailty index by checking whether it replicates certain regularities that have been established in the literature. A distinctive feature that has been stressed in previous studies is a strong and stable association between the log of the frailty index and age, implying that health deficits are accumulated at about a constant rate of 2.5 to 4.5 percent per year [7, 9, 19–23]. The quality of the index was demonstrated by its predictive power for mortality and other adverse health outcomes such as the risk of institutionalization in nursing homes and becoming a disability insurance recipient [7, 24–26]. Dalgaard et al. [9] estimated an elasticity of the mortality rate with respect to the frailty index of about 3.0 for a sample of (mostly) developed countries. The previous literature has also established that women, at given age, display more health deficits than men (see [27] for a review and meta study) while men develop new health deficits faster than women [20, 21, 28]. We therefore followed the literature and carried out the analysis separately for men and women.

The exponential growth of health deficits can be seen as the physiological expression of Gompertz law. Gompertz has shown that for people between the ages of 30 and 90 there is an almost perfect exponential increase in mortality with age [4, 29]. This “law” of mortality has been replicated in countless studies with an age coefficient of mortality estimated between 8 and 10 percent [30, 31]. The increase in mortality can be explained by increasing frailty. When health deficits increase at a rate of 3 percent per year (which is roughly the mean of the empirical estimates cited above) and the mortality elasticity of frailty is 3.0, the increase in frailty motivates a 3 × 3 = 9 percent increase of the mortality with age, consistent with Gompertz law.

Physiological Aging in India has been investigated by other studies before. Chatterji et al. [32] compare health of the aging population in India and China. From projections of disability-adjusted life years (DALYs) attributed to communicable and non-communicable diseases and population projections, they conclude that health of the elderly population will decline in the near future in India (but not in China). Bloom et al. [33] assess the macroeconomic costs of demographic change and health trends for five chronic diseases for China and India. Several studies reported the prevalence of chronic conditions and functional limitations and their association with socio-economic covariates for the elderly Indian population [34–36]. Harttgen et al. [37] and Biritwurm et al. [38] construct a frailty index for India as well as several other countries using WHO-SAGE data and explore its association with socio-economic covariates. Chaudhary and Arokiasamy [39] use the same data and report differences across six Indian states. Recently, data of the Longitudinal Ageing Study in India (LASI) was made public [40, 41]. These data provide rich information on individual health and household characteristics of the elderly Indian population. However, despite the name of the study, the data are currently only available for one wave. The cross-sectional nature of the data set makes it unsuitable for our longitudinal study with a focus on cohort analysis. The GBD-I data, by contrast, are ideally suited for the construction of frailty indices for cohort analysis at the level of Indian states. Dandona et al. [42] introduce the GBD-I data and report trends of prevalence of communicable and non-communicable diseases and DALYs for the Indian states grouped by their level of the epidemiological transition. None of the previous studies exploited a panel data set in order to identify the process of physiological aging of cohorts. We used the GBD-I data and constructed cohorts born between 1900 and 1995 and then investigated the aging of cohorts within states and over time and how the accumulation of chronic health deficits interacts with advances in the epidemiological transition.

## Methodology and data

### The frailty index

The frailty index, developed by Mitnitski and Rockwood and various coauthors, is a widely used measure for individual aging in gerontology and the medical sciences [5, 7, 23, 43]. It is constructed as the proportion of the total potential deficits that an individual has. The criteria for the selection of health deficits as items of the index are outlined in [44]: they need to be aging-related (prevalence increasing in age), associated with health status, not saturate too early, and cover a broad range of deficits. No specific deficit is required to enter into the index, since results appear to be unaffected by the specific list of deficits as long as a sufficient number of deficits—30 to 40—are included [7, 44].

Here, we follow the methodology developed in Dalgaard et al. [9] and compute the frailty index for populations. Specifically, the average frailty index of cohort *c*, gender *g*, in Indian state *s* is computed as $D_{cgs}=\frac{1}{P_{cgs}}\sum_{j}^{P_{cgs}}d_{jcgs}$, where *d*_*jcgs*_ is the frailty index of individual *j* from cohort *c*, gender *g*, living in state *s* and *P*_*cgs*_ is the number of individuals belonging to cohort *c*, gender *g*, living in state *s*. Using the definition of the individual frailty index, this expression can be re-arranged to obtain $D_{cgs}=\frac{1}{n}\sum_{a=1}^{n}\frac{P_{acgs}}{P_{cgs}}$, where *n* is the number of deficits included in the index and *P*_*acgs*_/*P*_*cgs*_ is the prevalence rate of age-related (disease) condition *a* in cohort *c*, gender *g*, state *s*. Therefore, in order to work out the aggregate frailty index for this particular age cohort, we simply need to calculate the average of *n* prevalence rates, *P*_*acgs*_/*P*_*cgs*_.

### Data and sampling

Data on disease prevalence rates are taken from the Global Burden of Disease study for India (Indian Council of Medical Research, 2017), henceforth cited as GBD-I [10], which covers the 29 Indian states and the Union Territory of Delhi (henceforth, cited as states) over the period 1990 to 2019. We have dropped the ‘residual state’ labeled Union Territories Other than Delhi, which combines the Andaman and Nicobar islands and 5 other small territories. The GBD-data is available only as an aggregate for these territories although these territories are scattered across India with vastly different geographic, social, and economic conditions, which cannot be controlled by state fixed effects in our panel analysis. In line with the literature on the frailty index, we included only adults aged 20 and above. The data base contains prevalence rates for men and women by five year age-groups. In the construction of the frailty index, we abided by the criteria listed in [44] and we strictly followed [9, 45] in the selection of disease items. This left us with 33 aging-related health conditions, which are listed in the S1 Appendix. The prevalence rates of these diseases are aggregated into the frailty index.

The age-group by year structure of the GBD-I data allows the construction of cohorts. For that purpose we kept every fifth year of observation and constructed 20 cohorts. The youngest considered cohort (cohort number 1) is 20–24 years old in 2019 and thus born born 1995–1999 (addressed as vintage 1995). The oldest cohort is 90–94 years old in 1990 (vintage 1900).

From the same database we also collected data on deaths from non-communicable diseases measured in deaths per 1000 persons (henceforth NCD mortality) and data on disability adjusted life years (DALYs) measured in DALYs per 1000 persons. Both variables are measured at the state and age-group level. Following Dandona et al. [42], we computed the epidemiological transition level (ETL) as the ratio of all-age DALYs due to communicable, maternal, neonatal, and nutritional diseases versus all-age DALYs due to non-communicable diseases. A high ETL value thus indicates an early stage of the epidemiological transition and a low ETL value an advanced stage.

We measured the level of socio-economic development by the Socio-Demographic Index (SDI), which is used in the GBD studies to account for demo-economic background [46]. The SDI computes three 0 to 1 indices for the total fertility rate under the age of 25 (TFU25), mean education for those ages 15 and older (EDU15+), and lag distributed income per capita (LDI), and then computes the geometric mean of the EDU15+, the LDI, and the inverse of the TFU25. The SDI at the state-by-year level is obtained from [47], see [48], pp. 1431–1433, for details on the computation of the SDI.

The summary statistics by state are shown in Table 1. A prominent feature is the relatively low variation of the frailty index within and across states. The total coefficient of variation is 0.54 for the frailty index versus 1.48 for NCD mortality. The mean frailty index ranges from 10.4 percent in Assam (and 7 other states) to 11.2 percent in Tamil Nadu. Across states a higher mean frailty index is associated with a greater standard deviation. The NCD mortality rate ranges from 30 in Haryana to 48 in Tamil Nadu. We consider the NCD mortality rate (rather than the crude mortality rate) because we want to explore the nexus between frailty and aging-related deaths. The NCD mortality rate is the unweighted average (across time and age) for the population in our sample (individuals 20 years and older) and it is measured in deaths per 1000 people in the state.

**Table 1:** Summary statistics by state.

State	Frailty Index		NCD Mortality		SDI		ETL	
	Mean	Std. Dev.	Mean	Std.Dev	Mean	Std. Dev	Mean	Std. Dev
Andhra Pradesh	0.108	0.059	33.337	45.925	0.410	0.088	1.004	0.453
Arunachal Pradesh	0.106	0.058	34.159	47.759	0.433	0.088	1.230	0.548
Assam	0.104	0.056	39.839	54.129	0.436	0.074	1.289	0.522
Bihar	0.104	0.057	31.483	44.210	0.329	0.065	1.813	0.720
Chhattisgarh	0.106	0.058	35.010	46.454	0.401	0.083	1.512	0.659
Delhi	0.109	0.060	39.082	56.884	0.601	0.074	0.821	0.360
Goa	0.107	0.059	37.083	55.651	0.614	0.075	0.460	0.226
Gujarat	0.106	0.058	38.578	58.762	0.481	0.084	1.084	0.459
Haryana	0.107	0.057	29.903	42.517	0.473	0.088	1.038	0.465
Himachal Pradesh	0.105	0.057	38.715	60.940	0.496	0.099	0.716	0.327
Jammu & Kashmir and Ladakh	0.104	0.057	39.023	58.475	0.471	0.094	0.867	0.389
Jharkhand	0.104	0.056	31.972	43.106	0.379	0.086	1.576	0.654
Karnataka	0.108	0.059	34.821	48.224	0.463	0.086	0.787	0.351
Kerala	0.109	0.062	42.237	71.359	0.551	0.082	0.290	0.118
Madhya Pradesh	0.104	0.056	33.680	46.550	0.387	0.071	1.697	0.710
Maharashtra	0.107	0.059	34.750	51.827	0.497	0.088	0.839	0.376
Manipur	0.105	0.057	34.987	49.912	0.493	0.070	0.913	0.333
Meghalaya	0.106	0.057	32.819	45.285	0.447	0.078	1.384	0.567
Mizoram	0.104	0.057	36.197	51.972	0.509	0.070	1.041	0.255
Nagaland	0.104	0.057	35.043	49.734	0.504	0.076	1.147	0.327
Odisha	0.106	0.057	30.700	42.822	0.411	0.083	1.397	0.589
Punjab	0.105	0.058	36.552	59.238	0.507	0.075	0.686	0.319
Rajasthan	0.104	0.056	35.546	52.266	0.381	0.090	1.733	0.734
Sikkim	0.107	0.059	35.591	51.111	0.486	0.104	0.798	0.427
Tamil Nadu	0.112	0.063	48.044	78.367	0.493	0.084	0.622	0.297
Telangana	0.107	0.058	39.608	59.216	0.427	0.094	1.025	0.474
Tripura	0.105	0.057	38.752	54.587	0.451	0.072	0.852	0.401
Uttar Pradesh	0.105	0.056	35.340	49.810	0.381	0.083	1.955	0.858
Uttarakhand	0.108	0.059	43.572	62.969	0.460	0.115	1.027	0.523
West Bengal	0.106	0.058	41.539	63.585	0.439	0.070	0.886	0.474
Total	0.106	0.058	36.599	54.120	0.460	0.104	1.083	0.633

**Fig 1:**
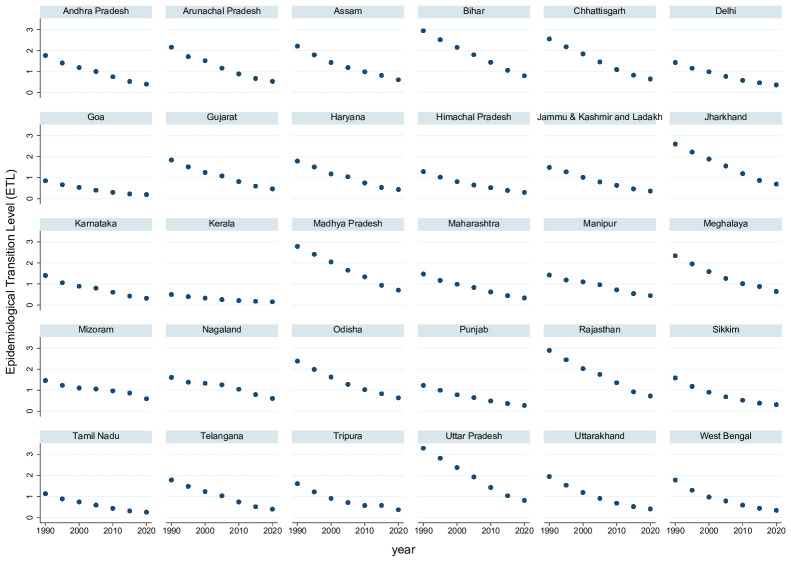
The indian epidemiological transition across states: 1990–2019. ETL is the ratio of all-age DALYs due to communicable, maternal, neonatal, and nutritional diseases versus all-age DALYs due to non-communicable diseases.

All Indian states experienced the epidemiological transition during the observation period, albeit with large differences in the initial level and in the speed of the process. Fig 1 shows the ETL-level from 1990 to 2019 for the 30 states. Initially, in 1990, the ETL level was above 2 in the states of Arrunachal Pradesh, Assam, Bihar, Chhattisgarh, Jharkhand, Madhya Pradesh, Meghalaya, Odisha, Rajasthan, Uttar Pradesh, and Uttarakhand. An ETL level above 2 indicates that communicable diseases contributed more than twice as much to the overall DALYs than non-communicable diseases. Only in Goa and Kerala the inital ETL level was below 1. In the end, in 2019, the ETL level was below 1 everywhere, but—with a minimum of 0.15 in Kerala and a maximum of 0.81 in Uttar Pradesh—far from accomplished convergence between states.

Notice that all states from the Empowered Action Group (Bihar, Chattisgarh, Jharkhand, Madhya Pradesh, Odisha, Rajasthan, Uttarakhand, and Uttar Pradesh) displayed an initial ETL level above 2, indicating that socio-economic backwardness is associated with backwardness in the epidemiological transition. Therefore, in our analysis of the effects of the epidemiological transition on aging, we control for the level of socioeconomic development through the SDI variable to prevent the coefficient of the epidemiological transition from picking up effects of economic development.

The particularly high average frailty index and NCD-mortality in Tamil Nadu cannot completely be explained by its high population share of elderly people. Other states that are similarly advanced in the epidemiological transition (such as Goa and Kerala) have similar or higher shares of elderly people ([41], Table 1.2) but lower average frailty and NCD-mortality. A potential reason for Tamil Nadu’s poor performance could be social isolation since the share of divorced, separated, and deserted individuals as well as the proportion of the elderly living alone are particularly high in Tamil Nadu ([41], p. 47, and Table 14.4, see [49] for the impact of social vulnerability on frailty, see [50] on the determinants of the living arrangements of elderly people in North and South Indian states).

## Results

### Age and aging

From previous studies we have a strong prior that health deficits are accumulated at about a constant rate, i.e. exponentially with age [9, 19–23]. In order to explore whether a similar pattern is observable in India, we regressed the logarithm of the frailty index (log_deficits) on a full set of age dummies, using all our available data, but estimated separately for each gender. Results are shown in Fig 1. Until about age 75, log deficits increase approximately linearly with age. Noting that an exponential accumulation of health deficits is represented by a linear progression of the estimation coefficients, we confirm for India the general pattern observed for individuals from Europe and North America [19–21, 23] and for populations across the world [9] that health deficits are accumulated at about a constant rate. At ages above 80, log deficits seem to approach a plateau in India. This feature is likely to reflect the survivor effect of particularly robust individuals that survived exposure to a high disease environment in their young and middle ages. A similar plateau effect is observed for mortality of the oldest old (ages above 90) in developed countries [51]. We also confirm for India the familiar gender pattern of aging found in the earlier studies: men start out somewhat healthier when young and then age faster than women, with alignment of health deficits around age 75.

In order to explore the aging process within states and within cohorts, we regressed the log frailty index on a linear age variable:

$$\begin{array}{c} {\text{log\_deficits}}_{asj}^{g}=\mu_{g}{\text{age}}_{sj}^{g}+\theta_{s}+\theta_{j}+\varepsilon_{asj}^{g}, \end{array}$$ (1)

in which *j* identifies either the year or the cohort and ${\text{log\_deficits}}_{asj}^{g}$ is the logarithm of the frailty index for age group *a* (20–24, ‥, 90–94) in state *s* observed either at year *j* (year 1990, 1995, ‥, 2019) in period analysis or for cohort *j* (cohort number 1,2,…,20) in cohort analysis. The variables are indexed with superscript *g* because we estimated (1) for each gender (female, male) in separate samples. State fixed effects are denoted by *θ*_*s*_ and *θ*_*j*_ denotes, respectively, year or cohort fixed effects. Since ${\text{age}}_{sj}^{g}$ is linear in age, the estimated coefficient *μ*_*g*_ quantifies the five-year growth rate of deficits (in age) for gender *g*. Dividing *μ*_*g*_ by 5 provides the growth rate of deficits by age at annuals levels. The error term is $\varepsilon_{asj}^{g}$.

Table 2 shows the regression results. In column (1) and (3) we control for state and year fixed effects, i.e. the results show the aging of populations within states, controlling for state and year specific characteristics (such as the level of the epidemiological transition). On average, Indian women accumulate 2.84 percent (0.142/5) additional health deficits from one birthday to the next. Men age slightly faster at 2.96 percent, but start with fewer deficits than women (indicated by the significantly smaller constant for men). The coefficients are estimated with great precision and comparable in size to the speed of aging found for individuals and (sub-)populations from other studies [9, 19, 21, 23]. In column (2) and (4), we control for cohort- instead of year-fixed effects. Within cohorts, aging takes place at a somewhat slower pace of 2.76 percent (0.138/5) health deficits per year for women and 2.90 for men.

**Table 2:** Aging in India.

	(1)	(2)	(3)	(4)
Gender	Women	Women	Men	Men
Age	0.142***	0.138***	0.148***	0.145***
	(0.000)	(0.001)	(0.000)	(0.000)
Constant	-3.523***	-3.489***	-3.651***	-3.623***
	(0.003)	(0.004)	(0.002)	(0.003)
Observations	3,150	3,030	3,150	3,030
*R* ^2^	0.970	0.986	0.971	0.984
State FE	Yes	Yes	Yes	Yes
Year FE	Yes	No	Yes	No
Cohort FE	No	Yes	No	Yes

The table reports results from estimating (1). The dependent variable is the log of the frailty index (log deficits). The age variable takes a separate value for each age group (ages 20–25 to 90–94). Standard errors, clustered at state level, are reported in parenthesis;

*** indicates statistical significance at the 1% level.

### Aging and mortality

**Fig 2:**
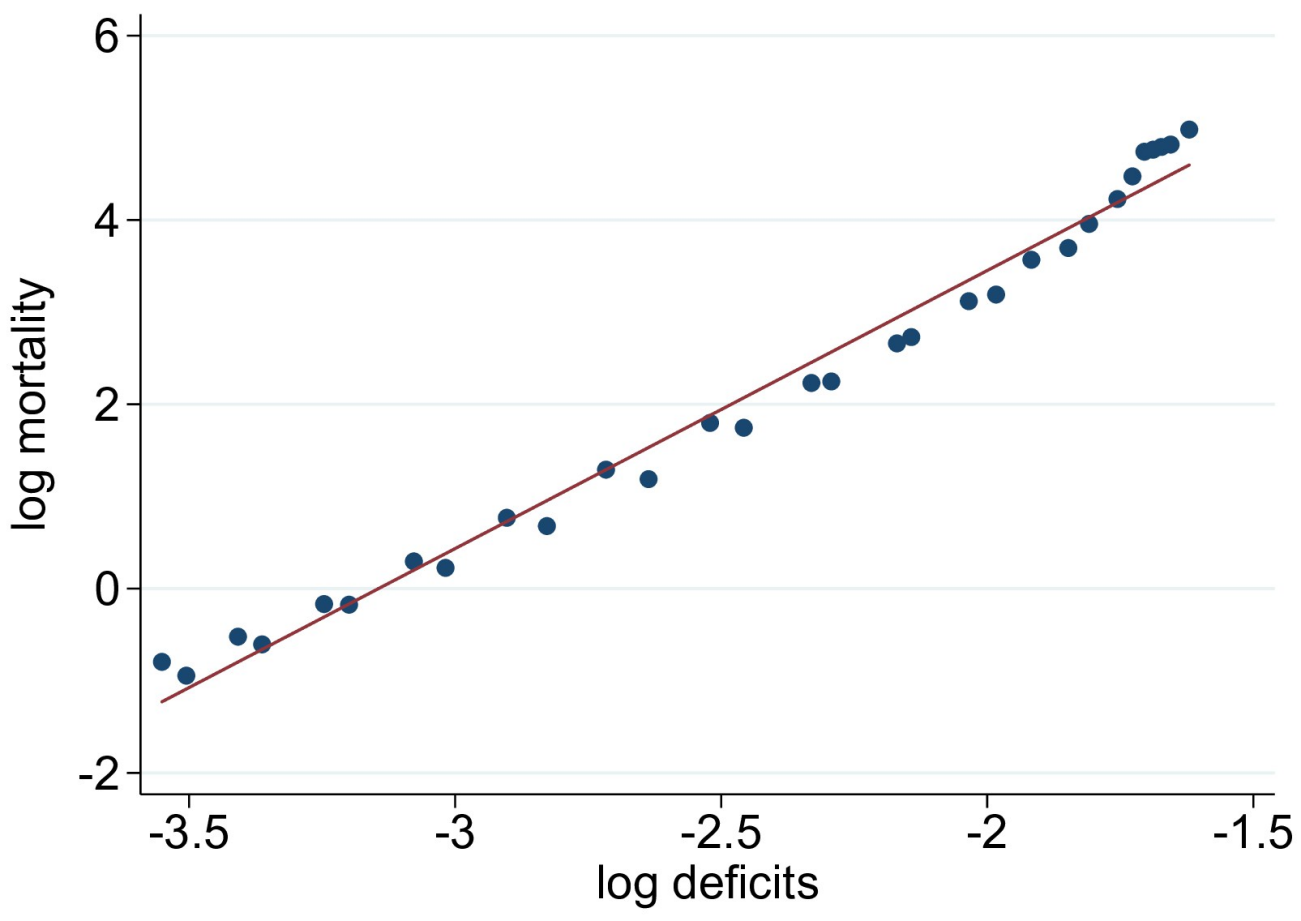
Frailty index and NCD mortality. Log deficits are grouped in 30 equally sized bins. The scatter plot shows the association of the mean log deficits and log mortality (deaths per 1000 adults) for each bin. The figure contains the fitted regression line.

As explained in the Introduction, previous research on aging in developed countries has found a strong association between frailty and mortality. In order to relate the Indian data to these studies, we focus on mortality from non-communicable diseases (NCD mortality). Fig 2 shows the ‘raw’ association between the frailty index and mortality for India. Log deficits have been sorted into 30 equally sized bins. The figure documents a strong association of mean log deficits and log NCD–mortality (deaths per 1000 adults) for each bin. In order to explore the morbidity–mortality nexus further, we estimated the following log-log relationship:

$$\begin{array}{c} {\text{log\_mort}}_{ast}^{g}=\beta_{g}{\text{log\_deficits}}_{ast}^{g}+\lambda_{s}+\lambda_{t}+\lambda_{a}+\epsilon_{ast}^{g}, \end{array}$$ (2)

where log_mort_*ast*^*g*^_ is the age-specific log of the NCD mortality rate in state *s* in period *t*. The fixed effects are given by the λs for state- (*s*), period- (*t*), and age- (*a*) fixed effects. The remaining variables are defined as above and we estimated *β*_*g*_ for each gender. Results are shown in Table 3. Column (1) and (5) show the raw correlation depicted in Fig 2, differentiated by gender. The precisely estimated coefficients do not change significantly when we control for state and age fixed effects (in columns 2–3 and 6–7). The estimates imply a power law association between mortality and the frailty index, *m* ∝ *d*^*β*^ with *β* = 3.1 for women and 2.9 for men.

**Table 3:** Frailty index and NCD mortality.

	(1)	(2)	(3)	(4)	(5)	(6)	(7)	(8)
Gender	Women	Women	Women	Women	Men	Men	Men	Men
log deficits	3.135***	3.137***	3.137***	2.051***	2.908***	2.909***	2.912***	1.668**
	(0.027)	(0.027)	(0.027)	(0.617)	(0.023)	(0.024)	(0.024)	(0.628)
Constant	9.493***	9.497***	9.497***	6.906***	9.491***	9.494***	9.501***	6.435***
	(0.053)	(0.064)	(0.064)	(1.472)	(0.055)	(0.058)	(0.058)	(1.547)
Observations	3,150	3,150	3,150	3,150	3,150	3,150	3,150	3,150
*R* ^2^	0.955	0.959	0.963	0.993	0.971	0.974	0.978	0.993
State FE	No	Yes	Yes	Yes	No	Yes	Yes	Yes
Year FE	No	No	Yes	Yes	No	No	Yes	Yes
Age FE	No	No	No	Yes	No	No	No	Yes

The table reports results from estimating Eq (2). The dependent variable is the log of the NCD mortality rate. Standard errors, clustered at state level, are reported in parenthesis; ** (***) indicates statistical significance at the 5% level (1% level).

Columns (4) and (8) show results when we additionally controlled for age fixed effects. The coefficient on log_deficits thus reflects the contribution of health deficits to mortality for given age. This exercise is interesting against the background that the mortality predictions in the demographic literature typically rely solely on age, for example, based on the Gompertz law. The estimates indicate that knowing the frailty index of individuals substantially improves mortality predictions. Comparing the coefficients from column (3) and (4) shows that, for women, 2/3 of the coefficient of health deficits cannot be explained away by knowing the age of an individual. For men, this ratio is somewhat lower at 57 percent, as shown by comparison of the coefficients from column (7) and (8).

### Aging and the epidemiological transition

**Fig 3:**
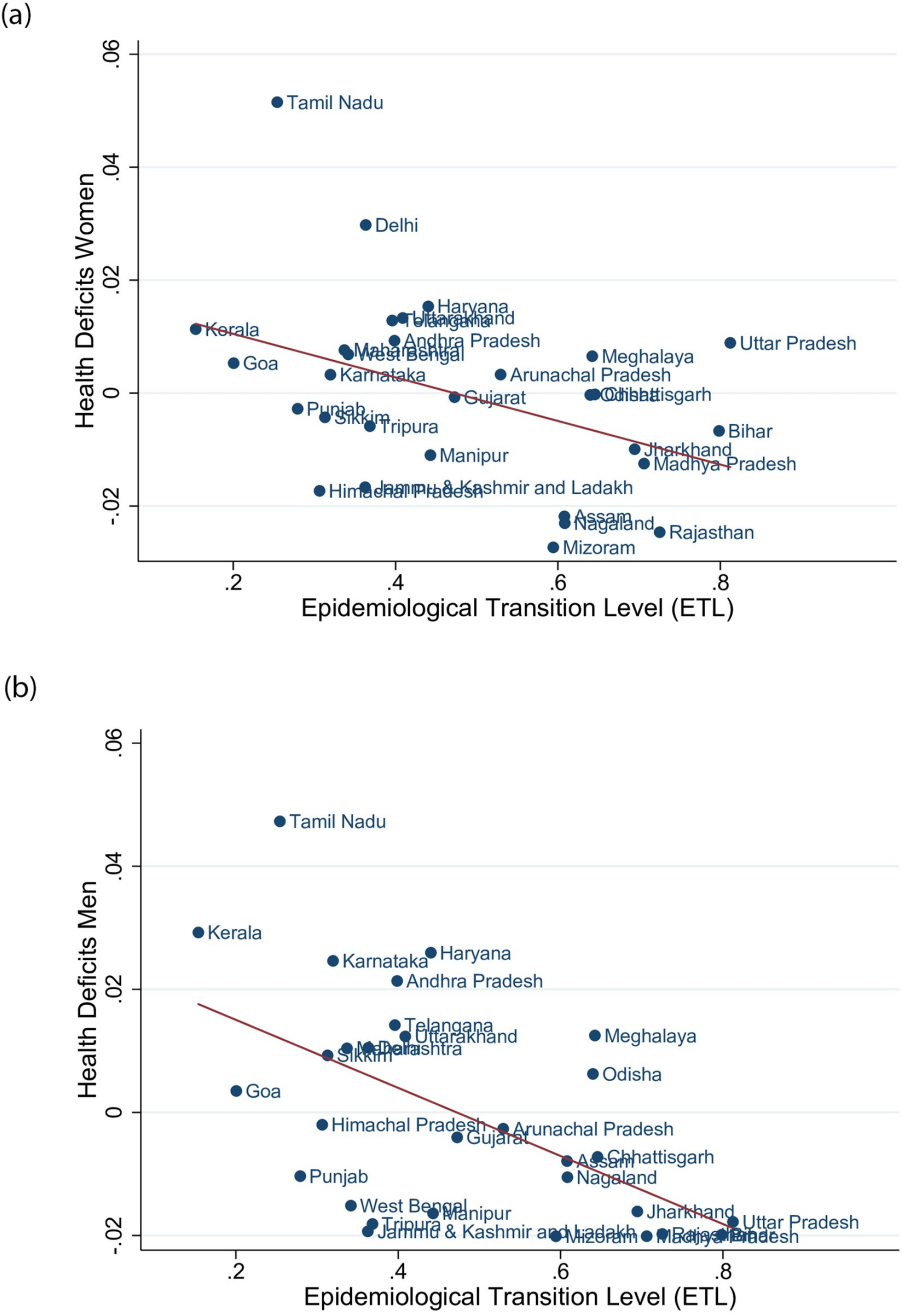
Epidemiological transition and health deficits: State fixed effects. The figures show the state fixed effects from the regressions reported in columns (2) and (4) of Table 2 against the ETL-value of 2019. The panel on the left hand side shows results for women, the panel on the right hand side shows results for men. The regression line is 0.0190 − 0.0386*ETL* for women and 0.0261 − 0.0553*ETL* for men.

We next explored the association of the aging process with the epidemiological transition. Fig 3 shows the state fixed effects from the regressions in columns (2) and (4) of Table 2 and relates them to the level of the epidemiological transition in 2019. The mean of the state fixed effects is zero (by construction) and the standard deviation is 0.016, implying a substantial location effect on health deficits. For example, for given age and cohort, women in Delhi display about 3 percent more health deficits than the Indian average. Fig 3 shows a negative association between health deficits and the ETL-level. On average, states at a more advanced level of the epidemiological transition (with lower ETL value) display more age-related health deficits. The slope of the regression line for men is −0.055, implying the prediction that when the epidemiological transition advances from ETL 0.8 (the level of Bihar) to ETL 0.2 (the level of Goa) the frailty index increases by −0.055 ⋅ (0.2 − 0.8) = 3.3 percent. For women the slope of the regression line is flatter, at −0.038, implying the prediction of a 2.3 percent increase of the frailty index when ETL advances from 0.8 to 0.2.

The reason for the on average (across time and age) negative association between the frailty index and the epidemiological transition is a selection effect (which we discuss in detail in the simulation section): at low ETL levels (i.e. more advanced epidemiological transition), more people survive to an old age and as chronic health deficits accumulate exponentially with age, the prevalence of chronic health deficits in the population increases. The population average, however, does not allow to draw inferences about the impact of the ETL level on individual aging. In order to verify this claim and to investigate the role of the epidemiological transition as a process, we included ETL-by-state-by-year values into the regression Eq (1). To ensure that the ETL-coefficient does not pick up other dynamics of socio-economic development, we also added the state and year dependent SDI values to the regression.

Results are shown in Table 4. Columns (1)–(4) show results for the full sample, stratified by gender and columns (4)–(8) show results for sample splits according to the state of the epidemiological transition. Results across cohorts are reported in columns (1) and (3). The other columns show within-cohorts results. In all regressions, we find a significantly positive association between socio-economic development and health deficits, which increases substantially in size once cohort fixed effects are added to the model. Taking the standard deviation of the SDI of 0.10 (reported at the bottom of Table 1), the estimates in columns (2) and (4) of Table 4 imply that a one standard deviation increase of SDI increases health deficits by 3 percent (0.30 ⋅ 0.1) for women and 2.7 percent (0.27 ⋅ 0.1) for men.

**Table 4:** Aging and the epidemiological transition.

	(1)	(2)	(3)	(4)	(5)	(6)	(7)	(8)
Gender	Women	Women	Men	Men	Women	Women	Men	Men
ETL Level	All	All	All	All	Advanced	Late	Advanced	Late
Age	0.142***	0.133***	0.148***	0.138***	0.140***	0.123***	0.139***	0.135***
	(0.000)	(0.003)	(0.000)	(0.002)	(0.004)	(0.005)	(0.001)	(0.003)
SDI	0.049***	0.301***	0.126***	0.270***	0.265**	0.521***	0.296***	0.348***
	(0.015)	(0.074)	(0.012)	(0.040)	(0.091)	(0.150)	(0.036)	(0.083)
ETL	0.011***	0.031***	0.004*	0.018***	0.068***	0.024***	0.036***	0.016***
	(0.002)	(0.006)	(0.002)	(0.004)	(0.009)	(0.008)	(0.006)	(0.005)
Constant	-3.557***	-3.618***	-3.713***	-3.710***	-3.682***	-3.634***	-3.744***	-3.726***
	(0.010)	(0.021)	(0.008)	(0.013)	(0.029)	(0.039)	(0.017)	(0.019)
Observations	3,150	3,030	3,150	3,030	1,515	1,515	1,515	1,515
*R* ^2^	0.970	0.986	0.971	0.984	0.987	0.985	0.985	0.983
State FE	Yes	Yes	Yes	Yes	Yes	Yes	Yes	Yes
Cohort FE	No	Yes	No	Yes	Yes	Yes	Yes	Yes

The table reports results from estimating Eq (1) augmented by ETL and SDI as covariates. The dependent variable is the log of the frailty index (log deficits). The age variable takes a separate value for each age group (ages 20–25 to 90–94). SDI is the index of socio-economic development; ETL is the level of the epidemiological transition. Standard errors, clustered at state level, are reported in parenthesis; ** (***) indicates statistical significance at the 5% level (1% level).

Turning to the coefficient of interest, we found a positive association between ETL and the frailty index. Recalling that ETL declines with advancing epidemiological transition, this means that, for *given age and state*, health deficits decline as the epidemiological transition progresses. Focussing on the within-cohort estimates from column (2) and (4), we see that health deficits decline by 2.0 percent (0.031 ⋅ 0.633) for women and by 1.1 percent (0.018 ⋅ 0.633) for men when ETL declines by one standard deviation (standard deviation as reported at the bottom of Table 1). Going from an ETL value of 3.3 (Uttar Pradesh in 1990) to 0.15 (Kerala in 2019), health deficits are predicted to decline by 0.018 ⋅ (3.3 − 0.15) = 9.7 percent for women and 5.7 percent for men.

The results from Fig 3 and Table 4 together show that the association of the epidemiological transition and the frailty index depends crucially on the point of view: results from averages across states are very different to results within states and controlling for (chronological) age. Once we control for age, the SDI level, and state fixed effects, the association between the ETL level and the frailty index becomes significantly *positive*, showing that individuals (within states, at any given age) are healthier at an advanced level of the epidemiological transition.

Columns (5)–(8) of Table 4 show results when the sample is split into forerunners and latecomers of the epidemiological transition. Similar to the methodology introduced by Dandona et al. [42] we consider states with an ETL value in 2015 below the median value of 0.51 as being at an advanced state of the epidemiological transition and the other states as being at a (relatively) late state of the transition. The results show that women from forerunner states age faster in physiological terms than women from late states. Per 5 years of chronological age they accumulate 14.0 percent more health deficits while women from latecomer states accumulate 12.3 percent more health deficits. The finding of faster aging at further advanced ETL does not contradict the ETL level results from columns (1) and (2). In fact, it replicates for the epidemiological transition the general phenomenon that more healthy individuals age faster (known as the compensation effect). The average frailty index in advanced states is 10.4 percent compared to 10.7 percent in latecomer states of the epidemiological transition. The results in columns (5) and (6) also show that the health of women in advanced states benefits more from further advancing epidemiological transition (larger ETL coefficient) and is less harmed by socio-economic development (lower SDI coefficient). Results for men are similar but the differences between high and low ETL states are more moderate.

### Trends of frailty by year of birth

**Fig 4:**
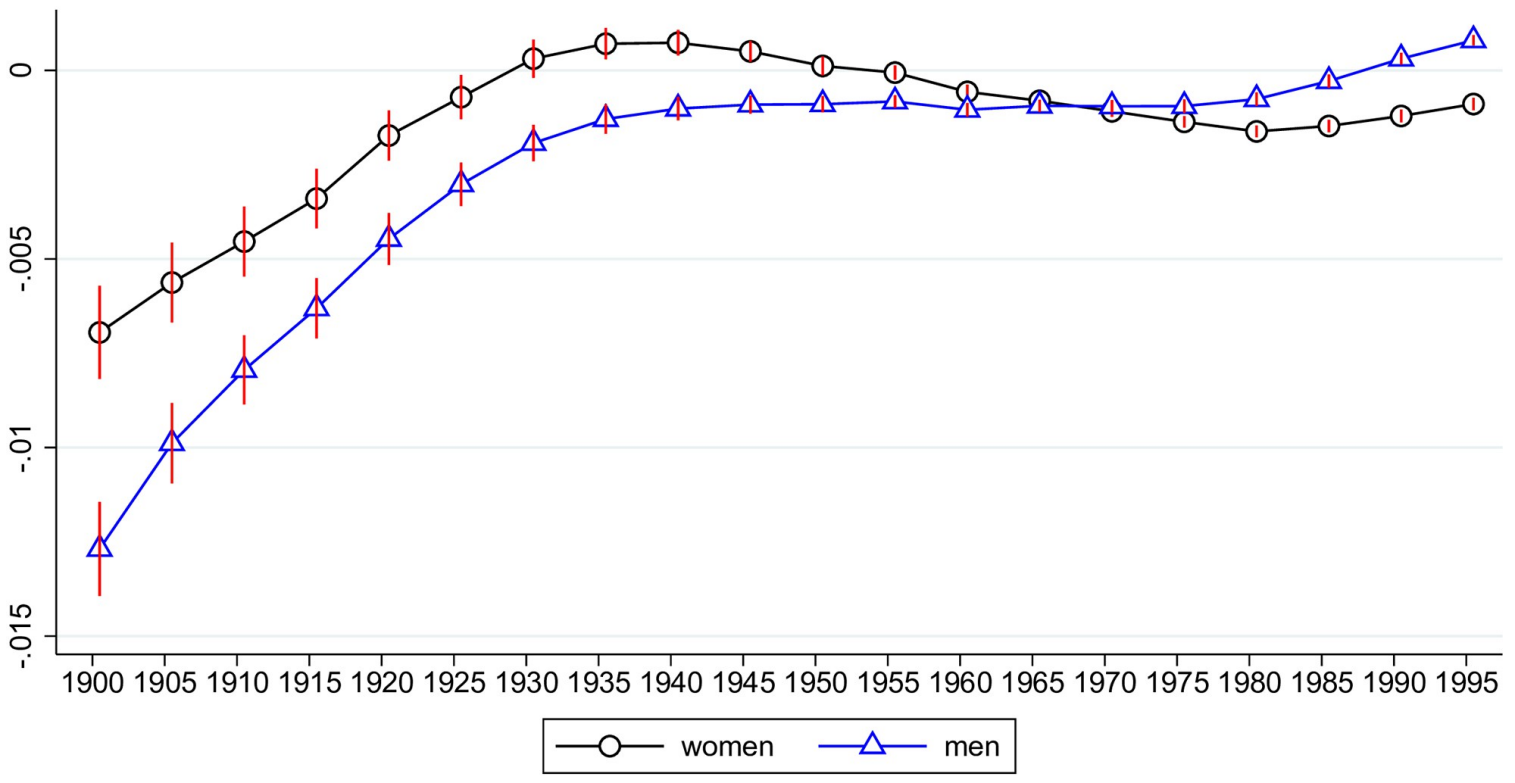
Trends of frailty by year of birth (controlling for age and state). The figure plots the estimates from regressing the frailty index on cohort dummies (indicated by birth years 1900, 1905, 1910, …), along with their 95% confidence bands. The regression controls for state and age fixed effects. Standard errors are clustered at the state level.

We next considered the aging of cohorts. For that purpose, we regressed the frailty index against a full set of cohort dummies, while controlling for age and state fixed effects. Results are shown in Fig 4. We see that later born cohorts display significantly more health deficits. The increase is driven by health deficits of individuals born before 1940. The cohort born 1940, is aged 50 in 1990 (when health deficits were observed for the first time) and 79 in 2019. When we split the sample at age-group 50–54, we found no or even a slightly improving health trend for individuals below 50 and a strong trend for those above 50, corroborating the conclusion that the trend of deteriorating health is driven by the earlier born generations.

**Fig 5:**
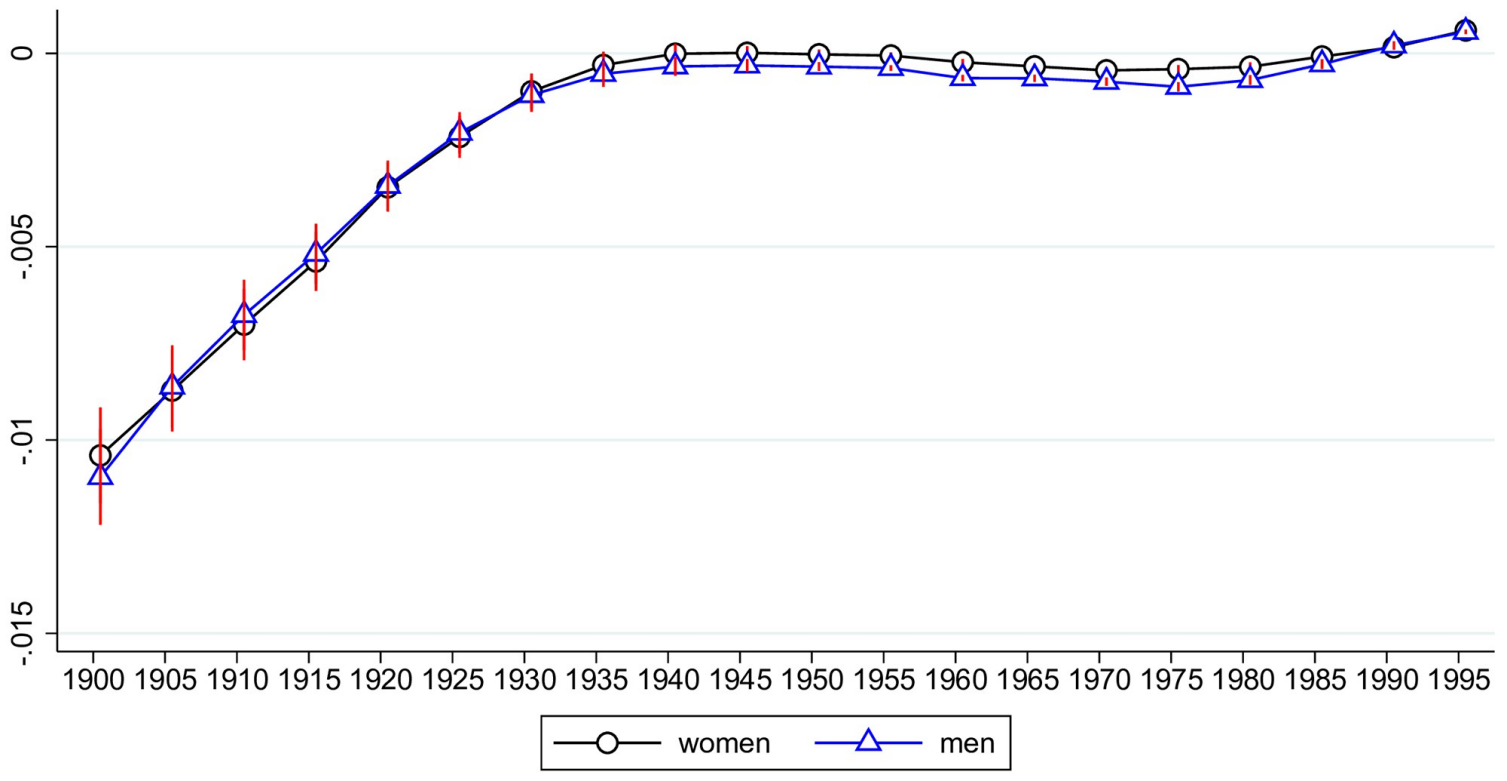
Trends of frailty by year of birth (controlling for age, state, ETL, and SDI). Estimates as for Fig 4, additionally controlling for the epidemiological transition (ETL) and socio-economic development (SDI).

In order to check to which extent these trends are driven by the level of the epidemiological transition and socio-economic development, we repeated the regression and additionally controlled for state-by-year values of ETL and SDI. The results are shown in Fig 5. We see that the cohort trends of frailty moved closer together. The trend for women differs only insignificantly from that of men. Also, there is almost no trend discernable for generations born after 1940. For generations before 1940, however, the trend remains substantial. Men and women born in 1940 have more than 20 percent of a standard deviation more age-related health deficits than those born in 1900 had at the same age.

## Aging during the epidemiological transition: A model

In this section, we show that the observed trend of increasing frailty across cohorts can be explained by a selection effect of the epidemiological transition. We consider a model-society in which any age-group consists of two types of individuals, indexed by *a* and *b*. Individuals are identical, aside from the fact that *a*-types started initially and for exogenous (e.g. genetic) reasons with more health deficits. Individuals accumulate deficits at a common rate such that, at any age, the *a*-type displays more health deficits. In the S1 Appendix, we formally prove that the average frailty index increases with advancing epidemiological transition if, due to the reduced exposure to infectious diseases, the survival probability of the unhealthy group (group a) increases relatively more than the survival probability of the healthy group (group b). Here we use results from a simulation of the model to illustrate how the observed trend for India may be motivated by a selection effect.

Without loss of generality, we focused on men and assumed that the two types of individuals accumulate health deficits at the same rate and differ in the initial deficits at age 20 such that *D*_*j*_(*t*) = *B*_*j*_e^*μt*^ for *j* ∈ *a*, *b*. Age and time are measured in years. For the calibration of the model, we set *μ* = 0.029 such that men accumulate health deficits at a rate of 2.9 percent per year of age, as estimated in Table 2. The survival probability is specified as *S*(*E*, *D*) = e^−*m*(*E,D*)^, in which *E*(*t*) measures the disease environment at time *t* (which is inversely related to the ETL level). The mortality rate is a log-linear function of the frailty index, log*m* = (1 + 1/*E*(*t*))*γ* + *ψ* log(*D*). We used the estimates from Table 3 to set *ψ* = 2.9 and assumed an inverse association of the shift parameter and the disease environment *E*(*t*).

**Fig 6:**
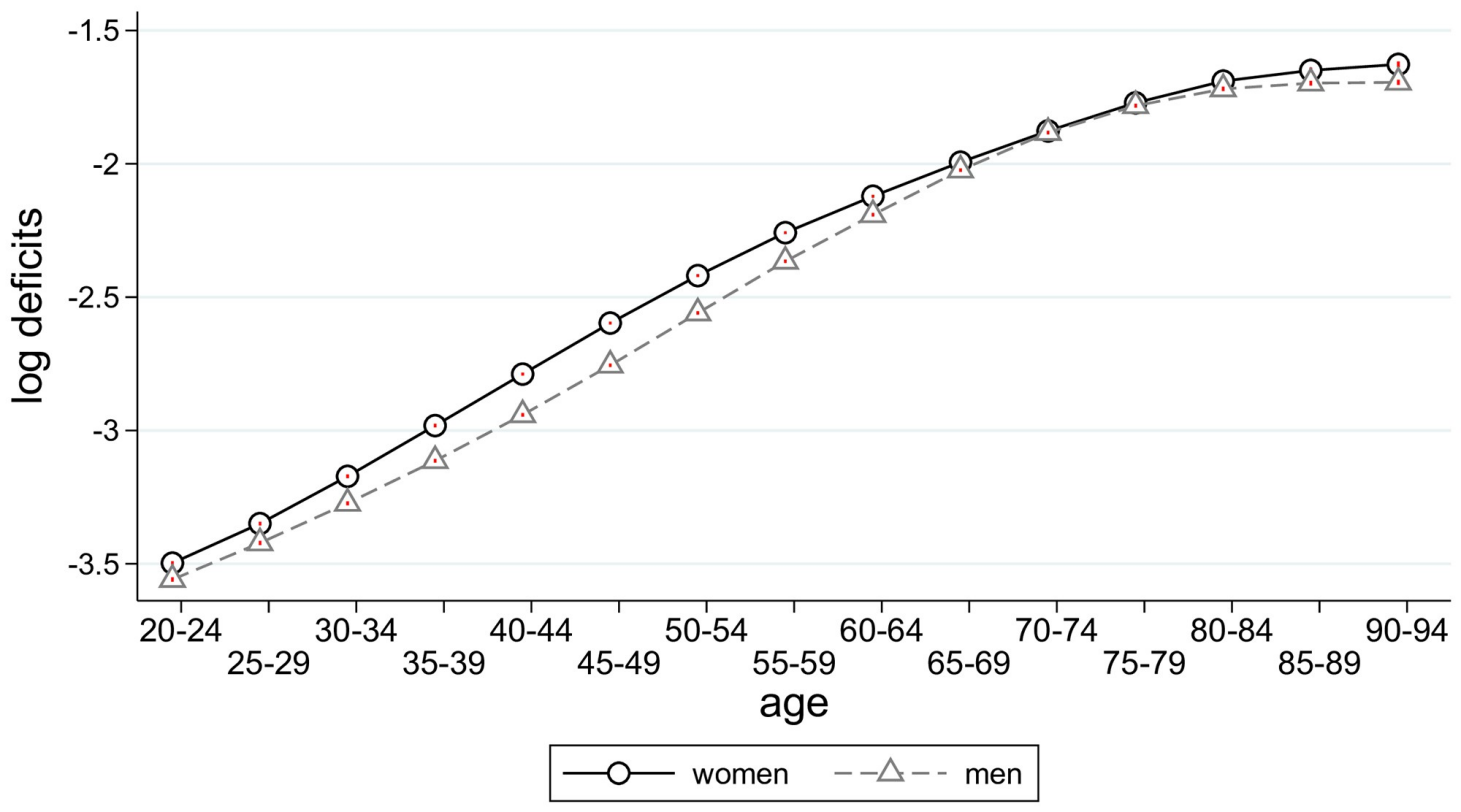
Average health deficits by age for women and men: 1990–2019. The figure plots the estimates from regressing log deficits on a full set of age-group dummies (omitting the regression constant) by gender, using data for all Indian states and all periods (1990, 1995, ‥ 2019), which corresponds to the average of log deficits for each age across countries and period.

**Fig 7:**
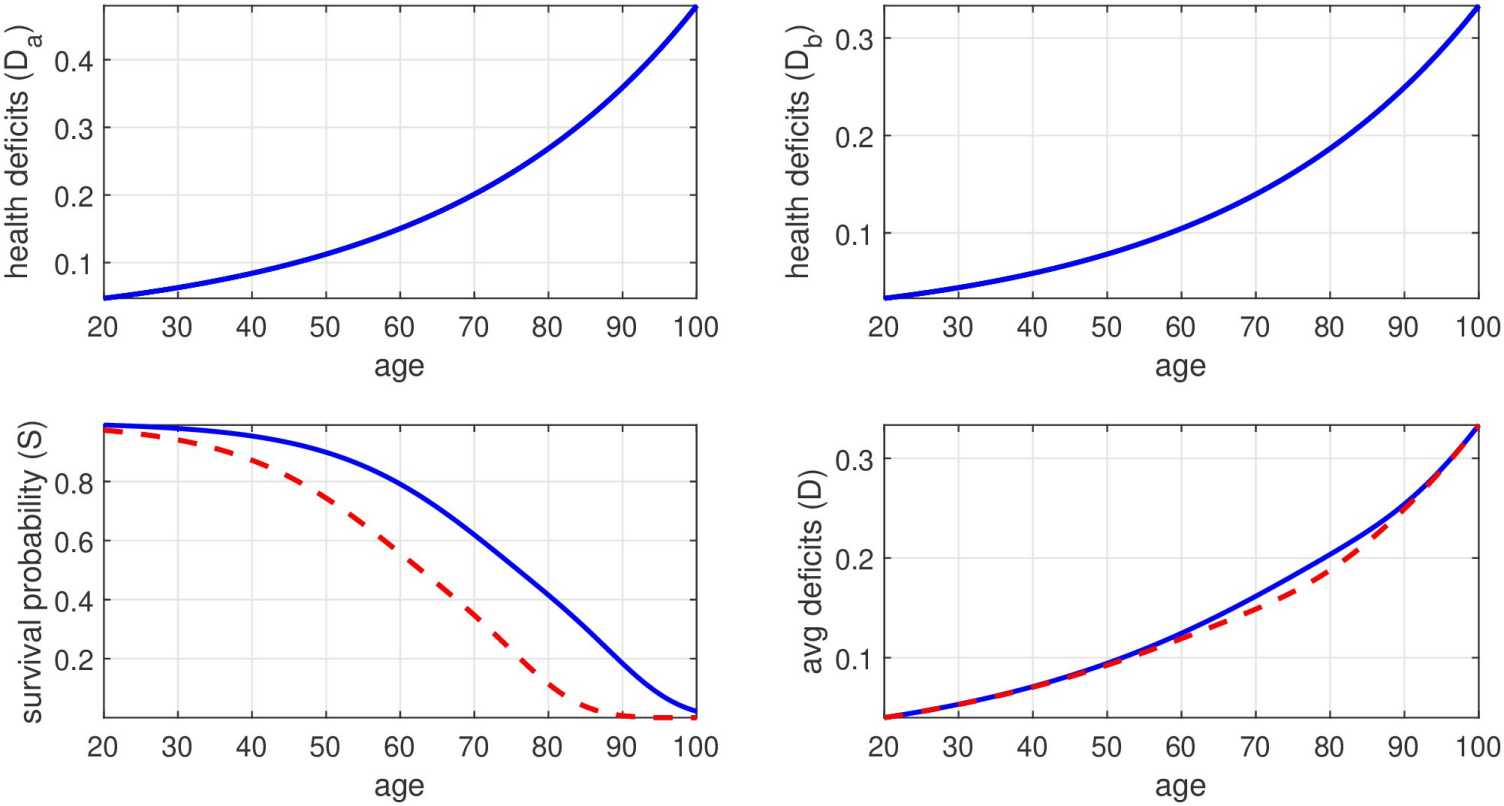
Simulation: Aging and the epidemiological transition 1990 vs. 2020. The figure shows in the upper panels the health deficits for initially less healthy (left) and initially healthy (right) individuals. The lower panels show the predicted average survival probability in the model society (left) and the predicted average health deficits by age (right) when the epidemiological transition is in an early phase (red dashed lines, *ETL* = 2.12) and in a late phase (blue solid lines, *ETL* = 0.52). Health deficits and survival probabilities are calibrated according to the estimates from Tables 2 and 3.

After running the model, we computed the implied life expectancy at 20 and used the WHO methodology [52] to compute DALYs, assuming a frontier life expectancy at 20 of 72 years. We also used the model to compute the contribution of communicable and non-communicable diseases to the DALYs and then computed the implied ETL value, which we related to the GBD-I data. Specifically, we calibrated the parameters *B*_*a*_, *B*_*b*_, *α*, and *γ* to match the following facts: a predicted life expectancy at 20 of 54 years, as reported [10] for India in 2019; an average frailty index of 0.2 at age 80 (see Fig 6); an initial frailty index that is 20 percent above average for group *a* and 20 percent below average for group *b*; an ETL value of 0.52, which is the Indian average in 2019. This led to the estimates *B*_*a*_ = 0.0264, *B*_*b*_ = 0.0183, *γ* = 80, and and *E*(2019) = 6.25. The predicted outcomes are shown by the blue solid lines in Fig 7. The upper two panels show the aging process of the two groups, the lower left panel shows the predicted survival curve, and the lower right panel shows average health deficits by age.

For the simulation experiment, we adjust *E*(1990) = 0.4 such that the predicted ETL value increases to 2.1, which was the Indian average in 1990. The predicted outcome is shown by red dashed lines in Fig 7. The lower left-panel shows the inward shift of the survival curve. The implied predicted life expectancy at 20 is 42, which somewhat underestimates the actual 1990 value. The predicted average health deficits are shown in the lower right panel. For elderly men, predicted health deficits in 1990 lie visibly below those from 2019. The largest difference in health deficits of 0.015 percent is observed at ages 70 to 80.

**Fig 8:**
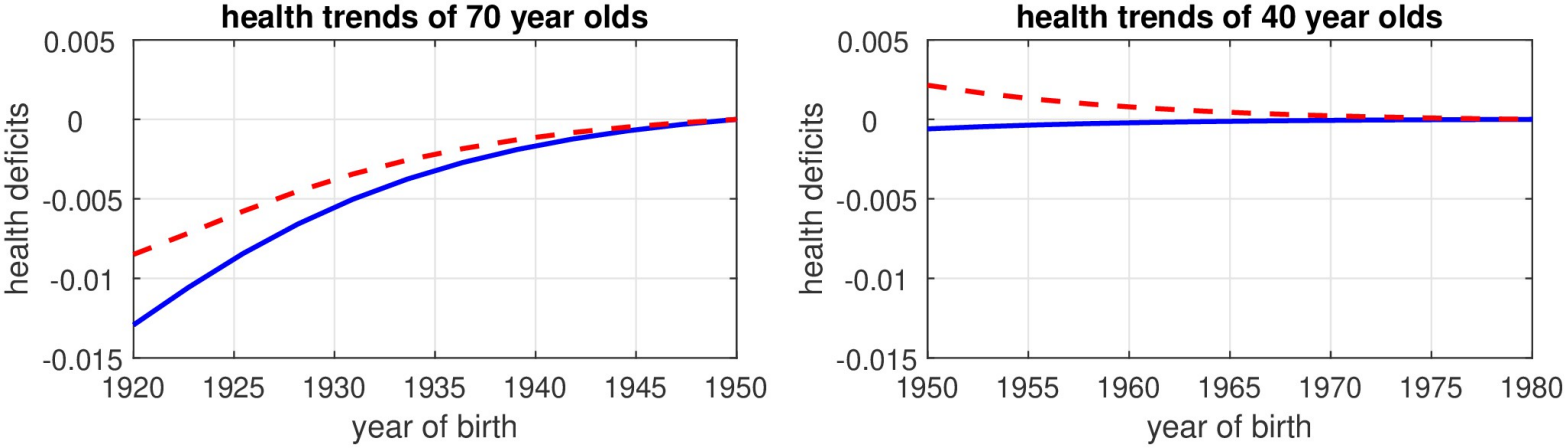
Simulation: Aging and the epidemiological transition from 1990 to 2020. The figure assumes that the ETL value declines at a constant rate from 2.7 to 1.1 as the calender year proceeds from 1990 to 2020. The panel on the left (right) hand side shows the predicted health deficits by year of birth for 70 year old (40 year old) men. Health deficits are measured in terms of deviation to the youngest cohort of the vintages shown in the panel. Dashed lines show predictions when a feedback effect from infectious diseases on chronic health deficits is taken into account. See text for details.

We next considered dynamics and simulated the epidemiological transition by assuming that the ETL value declined at a constant rate from 2.12 to 0.52 as time proceeded from 1990 to 2020. The predicted trends of health deficits are shown by blue solid lines in Fig 8. The panel on the left-hand side shows health deficits for 70-year-old men by year of birth. For example, in 1990 (when *ETL* = 2.12), 70-year-old men were born in 1920. Health deficits are measured relative to health deficits at the end of the transition. The calibrated model predicts a continuous decline of the health deficit difference, i.e. later born 70-year-old individuals are less healthy. The prediction also agrees in magnitude with the empirical estimates shown in Figs 4 and 5.

The panel on the right hand side of Fig 8 shows the trend of health deficits for 40-year-old men. Health deficits decline by later year of birth but the trend is minuscule compared to that of 70-year-old men. By putting the two panels together, we obtain a picture that resembles the overall trend result from Figs 4 and 5.

For the simulation, we assumed that health deficits from aging-related, non-communicable diseases are independent from the epidemiological transition. However, the evidence presented in Table 3 suggests that there is a feedback effect from infections to aging-related health deficits. In order to check robustness of results, we thus augmented the health deficit equation such that *D*_*j*_(*t*) = *B*_*j*_(1 + *θ*(1/*E*(*year*) − 1/*E*(2020))e^*μt*^). We took the point estimate from column (4) of Table 3 and the implied prediction that health deficits within cohorts decline due to the epidemiological transition by 3.8 percent from 1990 to 2020 (when the ETL value declined from 2.1 to 0.52). With this calibration target, we estimated *θ* = 0.017. The simulated health trends are shown by the red dashed lines in Fig 7. For 40-year-old men, results are non-robust to the model extension. Health deficits decline with later year of birth, albeit at a minuscule rate. For 70-year-old men, the trend of deteriorating health is robust and of similar magnitude as in the basic simulation. Altogether, the model thus supports selection due to infections as a mechanism that explains a negative health trend for later born generations of elderly people. For the young and middle aged there is no consistent trend. Both predictions agree with the results from Figs 4 and 5.

## Discussion

Previous studies have shown that, on average, individuals develop more health deficits at a nearly constant rate of between 2.5 and 4.5 percent per year of life [19–21, 23]. Recently, it has been shown that the physiological aging of nations progresses at a similar rate [9]. Here, we considered physiological aging of a developing country and confirmed the empirical regularity. Indian men and women accumulate age-related health deficits at a rate of 2.7 to 3.0 percent per year of life. The regularity is found across the population within states (i.e. controlling for state fixed effects and time fixed effects) as well as within cohorts born between 1900 and 1995. The exponential rise in health deficits reflects the self-productive nature of health deficit accumulation [53], i.e. the feature that the presence of many health deficits accelerates the development of new deficits [54]. We have also replicated for India the result from previous studies that men are initially (somewhat) healthier and age (somewhat) faster than women and the result of a power-law association between morbidity and mortality with an elasticity of about 3, suggesting that a one percent increase in the frailty index is associated with a three percent increase in NCD-mortality. The feature of faster aging of initially healthier people can be explained by applying reliability theory to biological aging; it has been expressed as the Strehler-Mildvan-correlation or the compensation effect of mortality [30, 55]. The feature that men are initially slightly healthier is consistent with a model were frailty depends on organ reserve and redundancy and the notion that on average larger men have more organ reserve [8, 56].

A unique feature of our study is the consideration of the physiological aging of cohorts along the epidemiological transition. Following Dandona et al. [42], we measured the epidemiological transition by the state-by-year ETL-value, which is the ratio between DALYs attributed to communicable diseases and DALYs attributed to non-communicable diseases. During the observation period there is substantial advancement of the transition (with an average ETL declining from about 2 to 1/2) as well as substantial variation across states (ranging from ETL values above 3 to values close to 1/10).

Focussing on aging *within cohorts*, we found a positive association of ETL and health deficits, implying that health deficits are on average smaller when the epidemiological transition is more advanced. The observation supports theories arguing that greater exposure to infectious diseases leads to faster development of chronic diseases [15, 16]. This interdependence is accounted for in a modern reformulation of the epidemiological transition hypothesis, which acknowledges that the phase of decline of infections is accompanied by a simultaneous decline in chronic diseases [12, 57]. For the U.S., for example, it has been shown that a large part of the decline in chronic diseases and functional limitations among older men from the early 20th century to the 1970s can be attributed to declining infections [13, 14]. The estimates of our study for India suggest that a decline from an ETL value of 3.3 (state of the epidemiological transition in Uttar Pradesh 1990) to 0.15 (Kerala in 2019) is associated with an average decline of aging-related health deficits of 9.7 percent for women and 5.7 percent for men.

However, comparing health deficits *across states*, we found that individuals in states at a more advanced stage of the epidemiological transition display *more* aging-related health deficits. Controlling for age and cohort fixed effects, the regression results predict that men in 2019 display on average 3.3 percent more health deficits in the most advanced states of the epidemiological transition compared to the most backward states. Similar but somewhat attenuated state fixed effects were found for women.

Investigating aging trends *across cohorts* born between 1900 and 1995, we found that later born cohorts display on average *more* health deficits. The benefit of an early year of birth is strongest among the oldest cohorts and disappears for cohorts born after 1940. The negative health trend is robust against controlling for state and age fixed effects, the advancement of the epidemiological transition, and socio-economic progress (measured by the SDI index) and it is of about the same size for men and women. The trend is small but precisely estimated. For example, Indians born 1905 displayed on average about one percent fewer health deficits than Indians born 1940 or later.

The observation of a mildly increasing frailty index is in contrast to Chang et al.’s finding that age-related health burden declined across the world at all levels of socio-demographic development [58]. It is more in line with Dalgaard et al.’s finding that health deficits by period of observation increased on average by about 2 percent from 1990 to 2019 in Asia, Africa, and the Americas (but not in Europe) [9]. The feature of a negative (for older generations) or absent (for younger generations) aging trend in India is furthermore in contrast to studies investigating the aging of cohorts of individuals in Europe and the USA, which found a trend of declining health deficits for later-born cohorts, which was interpreted as a result of effective medical technical progress [20, 59]. A distinguishing feature of the previous studies is their focus on advanced countries where the epidemiological transition ended well before the start of the observation period.

We thus finally explored whether the epidemiological transition can explain the increase of aging-related health deficits across cohorts in India. As discussed above, this trend cannot be explained at the individual level (or within cohorts) because there we found a positive association, in agreement with previous research on the nexus between exposure to infections and chronic diseases. Nor can the negative trend be explained by simple theories of complementarities and spillover effects between diseases [60]. According to these theories, a lower risk from communicable diseases (i.e. a more advanced state of the epidemiological transition) would induce more health investments in order to prevent and cure non-communicable diseases and should thus be associated with a lower frailty index at a given age.

In order to explain the aging trends in India, we proposed a model that generates a selection effect when cohorts composed of individuals with ex ante heterogeneous health status undergo an epidemiological transition. We calibrated the model with the estimated coefficients for India and derived a trend of increasing health deficits for the elderly population, explained by differential success of survival: relatively unhealthy individuals die young from infectious disease at an early stage of the epidemiological transition but survive to old age at an advanced stage of the epidemiological transition, thereby increasing the average health deficits of their cohort. This prediction is consistent with the assumption that all individuals at given age will become healthier during the epidemiological transition due to less exposure to infections and its effect on slower progression of chronic diseases. The selection-based theory is presumably also relevant for other countries during periods of fast advancement of the epidemiological transition and may motivate why improving health status at the individual level is not (yet) discernable at the cohort level.

A limitation of our study is that the structure of the GBD-I data does not allow for an examination of within-state variation in physiological aging or epidemiological transition. Our study also shares with other studies based on the GBD-I data several limitations concerning scarce and uncertain data for some locations and some diseases. These limitations were in detail discussed in [42, 46]. The frailty index is an appropriate strategy to ameliorate potential concerns. In particular, it has been shown that the index is very robust against the inclusion or omission of particular health deficits [44]. The main reason for this remarkable feature is that health deficits are partially (but not perfectly) correlated with each other such that missing or underreported information on certain items is taken up by correlated items [22]. Moreover, we validated the index for the Indian states by replicating distinct features of the index established in the previous literature.

In conclusion, our study has shown that physiological aging during the epidemiological transition is a complex phenomenon due to the interaction of individual health improvements and compositional effects. Depending on the underlying level of aggregation, we found the following results: At the highest level of aggregation, populations in states with a more advanced epidemiological transition have a higher frailty index. Within states and for a given age the frailty index improves as the epidemiological transition progresses. Across cohorts and for a given age, the frailty index worsens with the epidemiological transition, but only for cohorts constituting the elderly population in the 1990–2019 observation period. In this regard, our results for India differ from related studies in rich countries for which later born cohorts of elderly people have a lower frailty index. These health improvements are also likely to occur in India as the future population will grow up in an environment low in infectious diseases.

## Supporting information

S1 Appendix (PDF)
