# Supplementary material for: Physiological aging in India: The role of the epidemiological transition
Source: PLoS One. 2023 Jul 19;18(7):e0287259. doi: 10.1371/journal.pone.0287259 (PMC10355452; doi:10.1371/journal.pone.0287259)
Supplement: S1 Appendix — (PDF) [file pone.0287259.s001.pdf]

Physiological Aging in India: The Role of the Epidemiological Transition

Appendix

Astrid Krenz[1]
Holger Strulik[2]

[1] Ruhr University Bochum, Department of Management and Economics, Center for Entrepreneurship, Innovation and Transformation (CEIT), Universitaetsstrasse 150, 44801 Bochum, Germany
[2] University of Göttingen, Department of Economics, Platz der Göttinger Sieben 3, 37073 Göttingen, Germany; Correspondence to holger.strulik@wiwi.uni-goettingen.de.

A. Items in the Frailty Index. The frailty index is based on prevalence rates for the following diseases (33 in total):

Diarrheal diseases; Protein-energy malnutrition; Neoplasms; Ischemic heart disease; Stroke; Non-rheumatic valvular heart disease; Rheumatic heart disease; Hypertensive heart disease; Cardiomyopathy and myocarditis; Atrial fibrillation and flutter; Peripheral artery disease; Other cardiovascular and circulatory diseases; Chronic respiratory diseases; Peptic ulcer disease; Gallbladder and biliary diseases; Alzheimer's disease and other dementias; Parkinson's disease; Depressive disorders; Diabetes mellitus; Chronic kidney disease; Skin and subcutaneous diseases; Other sense organ diseases; Rheumatoid arthritis; Osteoarthritis; Low back pain; Gout; Urinary diseases and male infertility; Genital prolapse; Endocrine, metabolic, blood, and immune disorders; Oral disorders; Falls; Hearing loss; Blindness and vision loss.

Formal Derivation of the Selection Effect. Let $S(E(t), D_j(t))$ denote the probability of survival at age t, which depends and on age-related health deficits $D_j(t)$, with $j \in \{a, b\}$ and on the state of the epidemiological transition $E(t)$, which is exogenously given and the same for both types. An advancing epidemiological transition (i.e. a declining ETL value) is conceptualized as increasing E. Survival probability declines in the number of health deficits, $\partial S(E(t), D_j(t))/\partial D_j(t) < 0$ and increases with advancing epidemiological transition $\partial S(E(t), D_j(t))/\partial E(t) > 0$, reflecting the declining exposure to infectious diseases.

Suppose that both groups are initially of the same size, which is normalized to unity. Applying the law of large numbers, the average frailty index of any age-group t in the population is given by

$$D(t) = \frac{S(E(t), D_a(t))D_a(t) + S(E(t), D_b(t))D_b(t)}{S(E(t), D_a(t)) + S(E(t), D_b(t))}. \tag{1}$$

We then obtained the following result.

Proposition 1. *The average frailty index at age t increases with advancing epidemiological transition (increasing E) for age group t if*

$$\frac{\frac{\partial S(E(t), D_a(t))}{\partial E(t)}}{S(E(t), D_a(t))} > \frac{\frac{\partial S(E(t), D_b(t))}{\partial E(t)}}{S(E(t), D_b(t))}. \tag{2}$$

Proof: Differentiation of (1) with respect to E provides:

$$\frac{\partial D(t)}{\partial E(t)} = \frac{X - Y}{\left[S(E(t), D_a(t)) + S(E(t), D_b(t))\right]^2} \quad \text{with}$$

$$X \equiv \left[\frac{\partial S(E(t), D_a(t))}{\partial E(t)} D_a(t) + \frac{\partial S(E(t), D_b(t))}{\partial E(t)} D_b(t)\right] \left[S(E(t), D_a(t)) + S(E(t), D_b(t))\right]$$

$$Y \equiv \left[\frac{\partial S(E(t), D_a(t))}{\partial E(t)} + \frac{\partial S(E(t), D_b(t))}{\partial E(t)}\right] \left[S(E(t), D_a(t)) D_a(t) + S(E(t), D_b(t)) D_b(t)\right].$$

Thus, the frailty index increases in E for $X + Y > 0$. Collecting terms and simplifying, this condition can be expressed as

$$X - Y = \frac{\partial S(E(t), D_a(t))}{\partial E(t)} \left[S(E(t), D_b(t)) D_a(t) - S(E(t), D_b(t)) D_b(t)\right]$$
$$+ \frac{\partial S(E(t), D_b(t))}{\partial E(t)} \left[S(E(t), D_a(t)) D_b(t) - S(E(t), D_a(t)) D_a(t)\right] > 0,$$

which can be written in a more compact way as

$$X - Y = \left\{\frac{\partial S(E(t) D_a(t))}{\partial E(t)} S(E(t), D_b(t)) - \frac{\partial S(E(t) D_b(t))}{\partial E(t)} S(E(t), D_a(t))\right\} \left[D_a(t) - D_b(t)\right] > 0.$$

The term in square brackets is positive since $D_a(t) > D_b(t)$ by construction. The condition is thus fulfilled if the term in curly parenthesis is positive. This is the case for condition (2) stated in Proposition 1. qed.

The proposition shows that the average frailty index increases with advancing epidemiological transition if, due to the reduced exposure to infectious diseases, the survival probability of the unhealthy group (group a) increases relatively more than the survival probability of the healthy group (group b).
